# Cell Density-Dependent Upregulation of PDCD4 in Keratinocytes and Its Implications for Epidermal Homeostasis and Repair

**DOI:** 10.3390/ijms17010008

**Published:** 2015-12-23

**Authors:** Tao Wang, Shuang Long, Na Zhao, Yu Wang, Huiqin Sun, Zhongmin Zou, Junping Wang, Xinze Ran, Yongping Su

**Affiliations:** 1 Institute of Combined Injury, State Key Laboratory of Trauma, Burn and Combined Injury, Chongqing Engineering Research Center for Nanomedicine, School of Preventive Medicine, Third Military Medical University, Chongqing 400038, China; ls13648315854@163.com (S.L.); dunanadu@163.com (N.Z.); wangyusmmu@163.com (Y.W.); huiqinsun02@163.com (H.S.); wangjunping@tmmu.edu.cn (J.W.); ranxinze@163.com (X.R.); 2 Institute of Toxicology, School of Preventive Medicine, Third Military Medical University, Chongqing 400038, China; zmzou@tmmu.edu.cn

**Keywords:** programmed cell death 4 (PDCD4), keratinocyte, proliferation, epidermal homeostasis, wound healing

## Abstract

Programmed cell death 4 (PDCD4) is one multi-functional tumor suppressor inhibiting neoplastic transformation and tumor invasion. The role of PDCD4 in tumorigenesis has attracted more attention and has been systematically elucidated in cutaneous tumors. However, the normal biological function of PDCD4 in skin is still unclear. In this study, for the first time, we find that tumor suppressor PDCD4 is uniquely induced in a cell density-dependent manner in keratinocytes. To determine the potential role of PDCD4 in keratinocyte cell biology, we show that knockdown of PDCD4 by siRNAs can promote cell proliferation in lower cell density and partially impair contact inhibition in confluent HaCaT cells, indicating that PDCD4 serves as an important regulator of keratinocytes proliferation and contact inhibition *in vitro*. Further, knockdown of PDCD4 can induce upregulation of cyclin D1, one key regulator of the cell cycle. Furthermore, the expression patterns of PDCD4 in normal skin, different hair cycles and the process of wound healing are described in detail *in vivo*, which suggest a steady-state regulatory role of PDCD4 in epidermal homeostasis and wound healing. These findings provide a novel molecular mechanism for keratinocytes’ biology and indicate that PDCD4 plays a role in epidermal homeostasis.

## 1. Introduction

Programmed cell death 4 (PDCD4) is a tumor suppressor that has been implicated in the development of a broad spectrum of human tumors. PDCD4 was originally identified as a gene whose expression is induced in various types of apoptosis [1], and subsequently identified as a tumor suppressor in the JB6 mouse epidermal cell line model [2]. Then, experiments from both the PDCD4 transgene and knockout mice indicated that PDCD4 could inhibit cutaneous tumor incidence and papilloma-to-carcinoma conversion-frequency induced by TPA (12-*O*-tetradecanoylphorbol-13-acetate) [3,4]. Meanwhile, many reports showed that decreased expression of PDCD4 is associated with many kinds of tumors, such as tumors of the lung, breast, colon and liver [5,6,7,8]. These findings suggest that PDCD4 is a common tumor suppressor and plays an important role in carcinogenesis of a large spectrum of tumors.

Due to the important role of PDCD4 in tumors, the regulation of PDCD4 is the cause of much interest. The decrease in PDCD4 expression in many cases has been ascribed to the overexpression of microRNA miR-21, which can down-regulate PDCD4 at post-transcriptional level [9,10,11]. At the post-translational level, it has been reported that PDCD4 can be regulated by the ubiquitin-proteasome system [3,12]. In addition, PDCD4 can translocate between the nucleus and cytoplasm, and this kind of intracellular translocation may play an important role for tumor development [13,14,15]. As to mechanism research, a substantial body of evidence has suggested that PDCD4 functions by regulating other genes on two levels. PDCD4 affects transcription by inhibiting the activities of specific transcription factors including AP-1 and p53 [16,17]. Apart from that, PDCD4 acts as a suppressor of translation by interacting with and inhibiting the eukaryotic translation initiation factor eIF4A [18,19]. All these findings indicate that PDCD4 acquires the tumor suppressor properties by complicated regulation at multiple levels. Thus, PDCD4 could regulate critical events such as proliferation, differentiation, apoptosis and invasion in tumor progression [20,21,22,23].

Although PDCD4 was systematically elucidated as a tumor suppressor in various tumors, the normal biological function of PDCD4 is unclear. Especially in skin, although PDCD4 was originally identified as a tumor suppressor in mouse epidermal cell line model and in-depth studies of experimental oncology have been conducted in skin of genetically modified mice [2,3,4], the normal biologic role of PDCD4 in skin has not yet been revealed. Previously, Matsuhashi’s work [24] described the expression patterns of PDCD4 in human skin and some cutaneous tumors by immunohistochemistry, which suggested its role as homeostatic proliferation modulator of keratinocytes indirectly. While the exact role of PDCD4 in epidermal homeostasis and wound-healing is still unknown. Recently, we found that PDCD4 expression strongly increased in HaCaT keratinocytes upon achieving confluence by chance. Then, the function of PDCD4 in keratinocytes and its expression patterns in normal and wound epidermis were investigated. In this study, for the first time, we found that tumor suppressor PDCD4 is uniquely induced in a cell density-dependent manner in keratinocyte cells and serves as an important regulator of keratinocyte cell proliferation and contact inhibition *in vitro*. Also, knockdown of PDCD4 can induce upregulation of cyclin D1, one key regulator of cell cycle. Furthermore, enhanced expression of PDCD4 are detected in both anagen hair follicle and transient hyperproliferative wound epidermis *in vivo*, which suggests the a steady-state regulating role of PDCD4 in epidermal homeostasis and wound healing.

## 2. Results and Discussion

### 2.1. Programmed Cell Death 4 (PDCD4) Expression Depends on Keratinocytes Density

**Figure 1:**
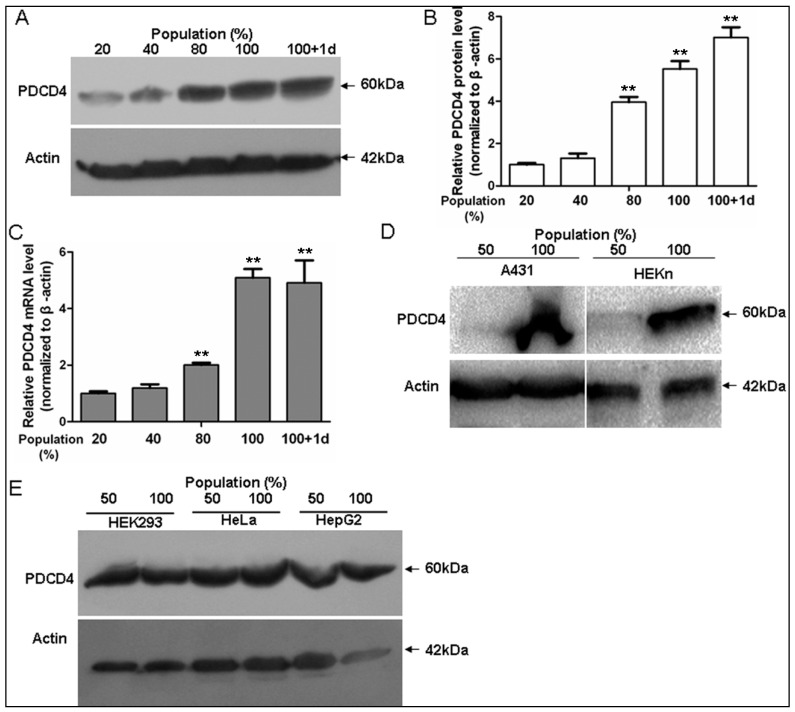
Upregulation of PDCD4 in human keratinocytes in a cell density-dependent manner. (**A**) Cell density-dependent expression of PDCD4 protein in human keratinocytes. HaCaT cells were cultured to reach the indicated cell densities. PDCD4 and β-actin expressions were analyzed by immunoblotting; (**B**) Densitometric analysis of PDCD4 expression at different cell densities. PDCD4 data were normalized to β-actin and plotted as means ± SD. The results shown are representative of three independent experiments; (**C**) Cell density-dependent expression of PDCD4 mRNA in HaCaT cells were determined by qRT-PCR (*n* = 3); (**D**) Cell density-dependent expression of PDCD4 protein in A431 human squamous carcinoma and HEKn primary human neonatal epidermal keratinocytes; (**E**) PDCD4 expression in other human cells. In HEK293, HeLa and HepG2 cells cultured to reach the indicated cell densities, PDCD4 was analyzed by immunoblotting. d, day; **, *p* < 0.01, when *vs.* 20% cell population.

It is reported that cell cycle arrest is induced upon high cellular density in keratinocytes, and the cell density-dependent upregulated p21 and HIF-α are closely associated with this process [25]. Similarly, we found the PDCD4 protein was induced in a cell density-dependent manner in HaCaT keratinocytes (Figure 1A,B). Also, the expression of mRNA level of PDCD4 was also induced by high cellular density detected by qRT-PCR (Figure 1C), which was in accord with the protein expression. Similarly, PDCD4 protein was also induced in both confluent A431 and HEKn cells, which are both derived from skin epidermis (Figure 1D). However, we did not find the upregulated PDCD4 at high cell density in non-keratinocyte cell lines such as HEK293, HeLa and HepG2 (Figure 1E). These findings indicate that PDCD4 is induced at the transcriptional level in a cell density-dependent manner in keratinocytes, and this kind of regulation is a unique feature of epidermal keratinocytes.

### 2.2. Knockdown of PDCD4 Promotes Keratinocytes Proliferation

**Figure 2:**
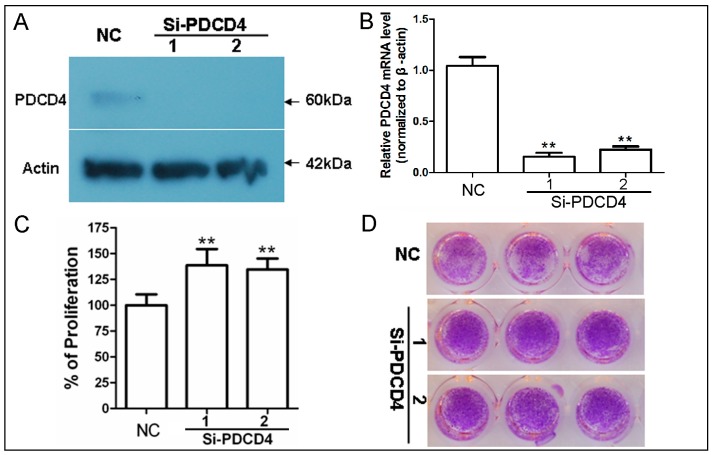
Knockdown of PDCD4 promotes keratinocytes proliferation. (**A**) Verification of PDCD4 knockdown in low-density HaCaT cells transfected with 50 nM siRNAs by western blot; and (**B**) real time PCR; (**C**) Proliferation assay in HaCaT cells transfected with PDCD4 siRNAs for 48 h with the CCK-8. Results represent the mean ± SD of three independent experiments. **, *p* < 0.001; (**D**) Crystal violet stain for clonogenicity capacity analysis of HaCaT cells silencing PDCD4 for 5 days in 96-well plate. NC, negative control siRNA. SD, standard deviation.

As cell density-dependent induced genes are always associated with growth arrest [25,26], then the role of PDCD4 in keratinocytes’ proliferation was investigated. We synthesized two short interfering RNAs (siRNA) for PDCD4 to determine the effect of PDCD4 knockdown on proliferation. The two siRNAs against PDCD4 sharply decreased PDCD4 expression in lower density (~20%) HaCaT cells (Figure 2A,B). The rates of cell proliferation in PDCD4 knockdown cells and control cells were subsequently analyzed using CCK-8 analysis. As expected, PDCD4 silencing significantly promoted HaCaT cells proliferation. The absorbance values of PDCD4 knockdown cells at day 2 were approximately 20%–30% higher than that of control siRNAs transfected cells (Figure 2C). A similar result was also seen at day 5 in gross observation of crystal violet staining (Figure 2D). These results demonstrated that knockdown of PDCD4 increases the rate of HaCaT keratinocytes proliferation, which indicate PDCD4 acting as important modulator of keratinocytes proliferation.

### 2.3. Knockdown of PDCD4 Impaires Contact Inhibition

**Figure 3:**
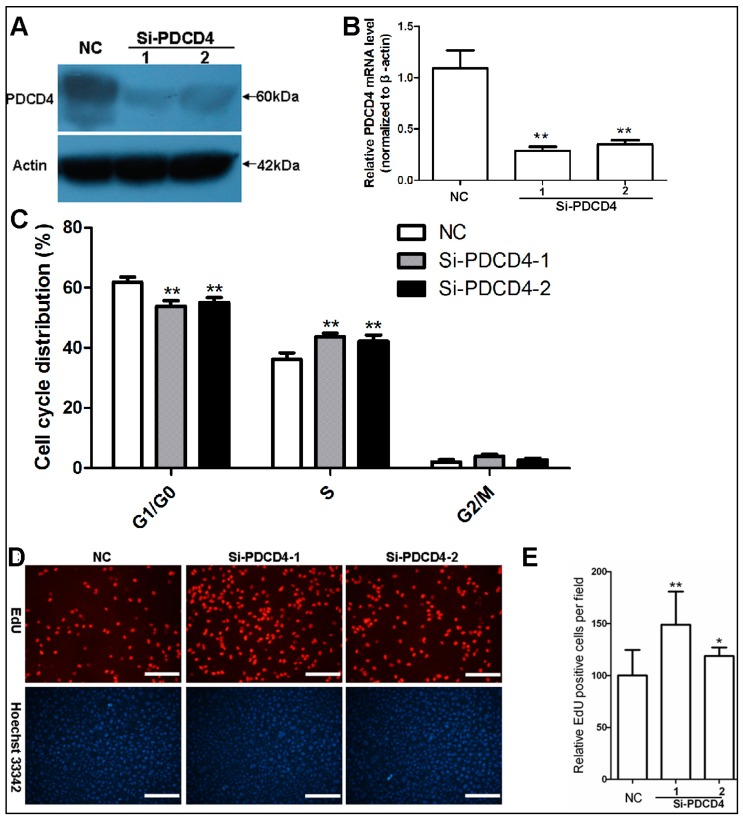
Knockdown of PDCD4 impaires contact inhibition. (**A**) Verification of PDCD4 knockdown in confluent HaCaT cells transfected with 50 nM siRNAs by Western blot; and (**B**) real time PCR; (**C**) Cell cycle analyses show weakened G0/G1 arrest of HaCaT cells transfected with PDCD4 siRNAs (*n* = 3); (**D**) Typical photos of the EdU assay are shown. Confluent HaCaT cells were transfected with PDCD4 siRNAs for 48 h, followed by 2 h incubation with EdU. Scale bar = 200 μm; (**E**) Quantification of EdU-positive cells in confluent HaCaT cells (*n* = 10). **, *p* < 0.01; *, *p* < 0.05. NC, negative control siRNA.

Tumor suppressor PDCD4 shows a cell density-dependent induced expression in HaCaT keratinocytes and has key role in proliferation, which led us to investigate the connection between PDCD4 and the establishment of the contact inhibition response. As shown in Figure 3A,B, siRNAs against PDCD4 sharply decreased PDCD4 expression in confluent HaCaT cells. The confluent cultures of PDCD4 silenced keratinocytes contained fewer cells in the G0/G1 phases of the cell cycle, along with more cells in the S and G_2_/M phases, compared with cultures of control siRNA transfected cells (Figure 3C). Furthermore, 5-ethynyl-2′-deoxyuridine (EdU) incorporation assay indicated that confluent cultures of PDCD4 knockdown groups had more EdU-positive proliferating cells than that of control cultures (Figure 3D,E). These findings suggest cell density-dependent induction of PDCD4 is required for keratinocytes’ contact inhibition.

### 2.4. Knockdown of PDCD4 Induces Cyclin D1 Expression

**Figure 4:**
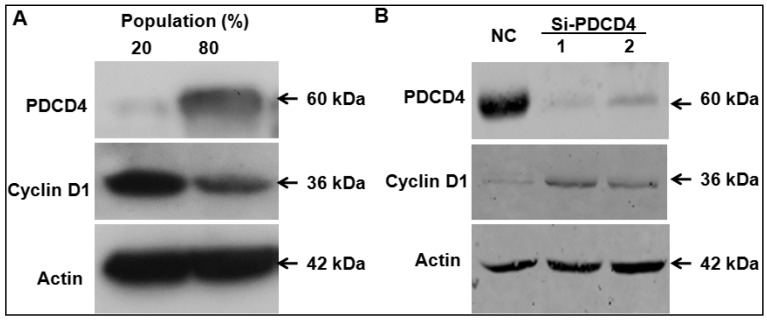
Knockdown of PDCD4 induces cyclin D1 expression in HaCaT cells. (**A**) Cyclin D1 expression was reduced in higher HaCaT cell density along with induction of PDCD4; (**B**) The protein level of cyclin D1 in PDCD4 silencing confluent cells were examined using Western blot. NC, negative control siRNA.

It has been reported that PDCD4 can regulate colon cancer cell line proliferation by modulating cyclin D1 [20], which is also one key regulator of keratinocytes’ proliferation[27]. The results of cell cycle analysis (Figure 3B) suggested that knockdown of PDCD4 shortened the G1 phase progression, which is similar to the results of knockdown of PDCD4 in HT29 cells [20]. We found that expression of cyclin D1 was reduced in higher cell density along with induction of PDCD4 (Figure 4A). Then, we tested whether knockdown of PDCD4 up-regulates cyclin D1 expression. As shown in Figure 4B, cyclin D1 protein level in PDCD4 silencing confluent HaCaT cells were induced obviously comparing to the control cells (Figure 4B).

### 2.5. Intracellular Translocation of PDCD4

**Figure 5:**
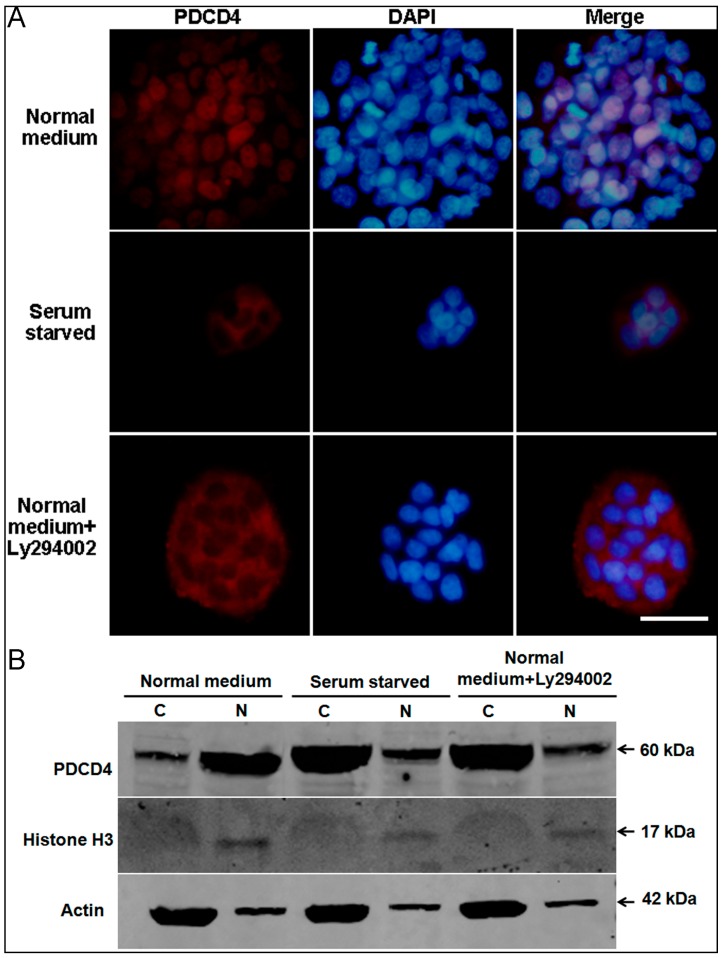
Intracellular translocation of PDCD4 in HaCaT cells. (**A**) Endogenous PDCD4 in HaCaT cells was localized exclusively in the nucleus when cultured in medium with serum (**top panel**). PDCD4 translocated from nucleus to cytoplasm under serum starvation treatment overnight (**middle panel**). In medium with serum, cytoplasmic localization of PDCD4 was observed, when cells treated with PI3K inhibitor Ly294002 (**bottom panel**). Scale bar = 30 μm; (**B**) PDCD4 was detected by Western blot of both cytosolic (C) and nuclear (N) protein fractions of HaCaT under different treatment conditions.

It is reported that PDCD4 functions in both the nucleus and the cytoplasm involving in the regulation of transcription and translation [13,15,28,29]. In epidermal cells, PDCD4 localized in nucleus of human specimens [24], but in cytoplasm of mouse epidermis [4]. As there are obvious histological differences between human and mouse epidermis [30], the different subcellular localization of PDCD4 seems to indicate different functional meaning. Then, the endogenous PDCD4 localization and its regulation were investigated in HaCaT human keratinocytes. As expected, PDCD4 showed clear nuclear localization under normal culture conditions detected by immunofluorescence (Figure 5A top panel). Of note, the fluorescence intensity in the central of the colony was stronger than that in the periphery, which was in accord with the cell density-dependent induction of PDCD4 protein as described in Figure 1. After serum starvation treatment for 48 h, PDCD4 localization became mostly cytoplasmic, confirming the published results [13] (Figure 5A middle panel). Study by Palamarchuk *etc.* [15] indicated that the intracellular localization of PDCD4 is regulated by Akt phosphorylation in the model of NIH-3T3 cells transfected with exogenous PDCD4. We then assessed the effect of Akt phosphorylation on the intracellular distribution of PDCD4 by treating normal cultrued HaCaT keratinocytes with PI3K inhibitor Ly294002. As shown in the bottom panel of Figure 5A, PDCD4 was redistributed to the cytoplasm when Akt phosphorylation was inhibited. To further demonstrate the intracellular translocation of PDCD4 between nucleus and cytoplasm, cytosolic and nuclear fractions of total protein were isolated and Western blot was performed to detect PDCD4. As shown in Figure 5B, PDCD4 was mainly located in nucleus under normal medium culture, while it translocated to cytoplasm upon serum starvation and Ly294002 addition. These findings indicate that PDCD4 could achieve intracellular translocation between nucleus and cytoplasm under stress and Akt phosphorylation, which suggests that PDCD4 could regulate transcription and translation interchangeably under certain circumstances.

### 2.6. PDCD4 Is Expressed in the Epidermis and Associated with Epidermal Homeostasis

**Figure 6:**
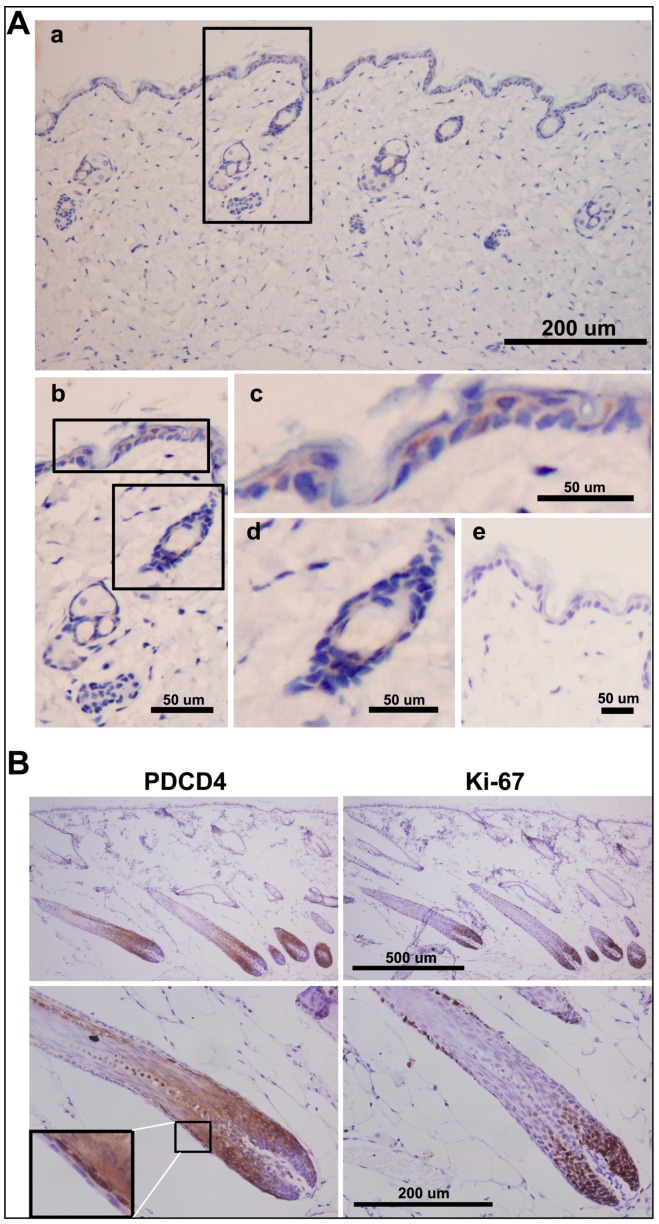
PDCD4 expression and its potential role in epidermis. (**A**) PDCD4 expression in normal mouse skin analyzed by immunohistochemistry. PDCD4 presented diffuse cytoplasmic staining in epidermal cells of interfollicular epidermis, telogen hair follicle and sweat gland (**a**); Partial enlarged detail (**b**); PDCD4 was stained discontinuously in both the suprabasal cells and basal cells of the interfollicular epidermis (**c**); PDCD4 was stained heterogeneously in the telogen hair follicle (**d**); A negative control was presented (**e**); the black box in (**a**) shows the source of the partial enlarged area of (**b**); the black boxes of (**b**) show the sources of partial enlarged drawings of (**c**,**d**); (**B**) Enhanced expression of PDCD4 in the anagen hair follicle. **Top**, there is mutual inverse expression of PDCD4 (**left**); and Ki-67 (**right**) in the anagen hair follicle (anagen VI, day 12 after hair growth induction by depilation) as shown at low magnification (×100); **Bottom**, immunohistochemical localization of enhanced PDCD4 expression in the anagen hair follicle (high magnification, ×400, **left**); Localization of Ki-67 in the anagen hair follicle detected by serial section (×400, **right**).

The function of PDCD4 in regulating keratinocytes proliferation and contact inhibition *in vitro* suggested its importance in the control of epidermis homeostasis *in vivo*. To explore the possible role of PDCD4 in epidermal homeostasis, the expression patterns of PDCD4 in epidermis were examined by immunohistochemistry. In specimens of human normal skin, PDCD4 was expressed in epidermis exclusively, and fibroblasts did not reveal any PDCD4 immunoreactivity. In epidermis, PDCD4 was stained diffusely in the suprabasal cells and discontinuously in the basal cells featured by heterogeneously nucleus localization (Figure S1), confirming the previously published results [24]. In mouse skin, PDCD4 was expressed in the interfollicular epidermis, telogen hair follicle and sweat gland but the signals were very weak. PDCD4 displayed diffuse cytoplasmic staining and apparent lack of nuclear staining (Figure 6Aa,b) as reported by others [4]. In the interfollicular epidermis, PDCD4 was stained discontinuously in both the suprabasal cells and basal cells (Figure 6Ac). In the telogen hair follicle, PDCD4 was stained heterogeneously in similar intensity as the interfollicular epidermis (Figure 6Ad). However, in the anagen hair follicle, PDCD4 was strongly induced and presented positive in both cytoplasm and nuclei of most stained cells (Figure 6B, left panel). The strong PDCD4-positive signals localized mainly in the cells of hair shaft before cornification and inner root sheath (IRS) surrounding hair shaft. While, the dermal papilla, germinative cell layer and outer root sheath (ORS) were PDCD4-negative (Figure 6B, left panel). Of note, the cells of hair shaft and the cells of IRS nestled up directly to ORS showed obviously strong nuclear staining (Figure 6B, left panel, partially enlarged detail). In contrast, there is mutual inverse expression of PDCD4 and Ki67 in the anagen hair follicle (Figure 6B, right panel). The Ki-67-positive cells localized mainly in the area without PDCD4 signals by staining serial sections. Taken together, the expression patterns of PDCD4 in epidermis suggest its potential role in maintaining epidermal homeostasis by modulating the hair follicle cycle.

### 2.7. Dynamic Changes in PDCD4 Expression during Epidermal Wound Healing

**Figure 7:**
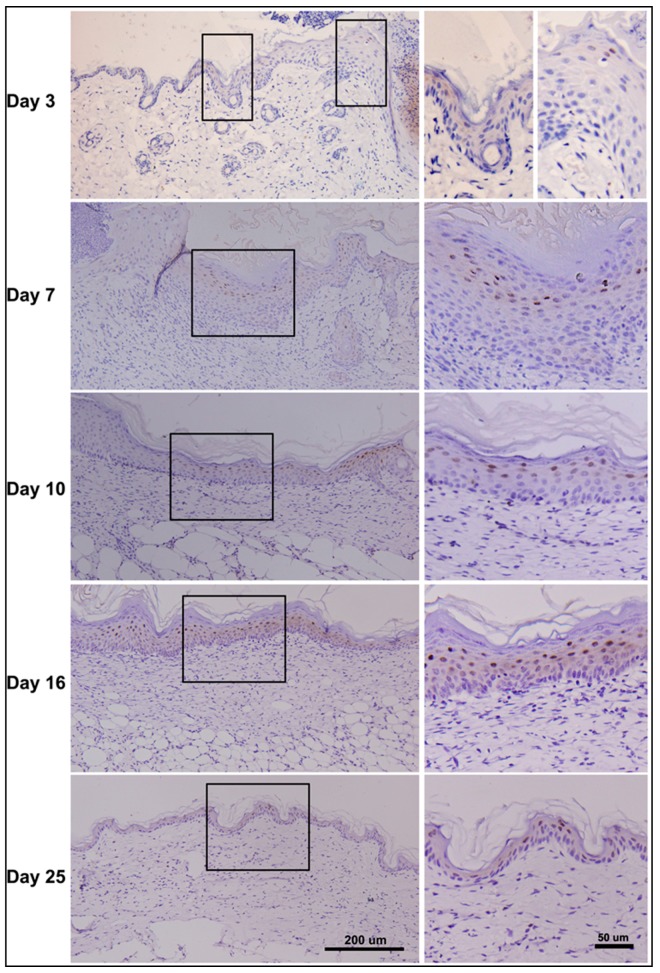
PDCD4 expression during cutaneous wound healing. Full thickness skin incision wounds were created on the backs of male BALB/c mice. The skin wound tissues were collected on the indicated days after wounding and the PDCD4 expression patterns were examined by immunohistochemistry (**left panel**); Partially enlarged drawings were presented (**right panel**); the black boxes (**left panel**) show the sources of the enlarged drawings (**right panel**).

The expression patterns of PDCD4 in normal epidermis combined with its negative regulatory role in keratinocytes proliferation suggest that PDCD4 plays an important role in regulating epidermal homeostasis. As both wounds and cancer tissue have highlighted remarkable similarities with common cellular and molecular mechanisms, we speculate that the tumor suppressor PDCD4 may also play roles in the process of reepithelialization. To investigate this possibility, expressions of PDCD4 during skin wound healing were examined by immunohistochemistry. We created full thickness incisional skin wounds on the backs of mice, and skin samples of the wounded area were collected at various times after wounding and stained with PDCD4 antibody (Figure 7). In the three-day wound section, slightly increased cytoplamic expression of PDCD4 was easily found in the starting position of the hyperplastic epidermis. As expected, significant loss of PDCD4 expression occurred in wound margin renewed epidermal cells besides a few suprabasal nuclear-positive cells. Many more PDCD4 nucleus-stained suprabasal keratinocytes emerged in the hyperproliferative epidermis but no signals were detected in the margin migratory keratinocytes in the 7-day and 10-day wound sections. While in the complete covered neo-epidermis of 16-day wound, both strong PDCD4 nuclear and cytoplasmic positive keratinocytes emerged in the whole suprabasal epidermis. Even in the thinner neo-epidermis of the wound after 25 days, the double nuclear and cytoplasmic positive keratinocytes persistently appeared. Thus, the dynamic intensity and localization expression changes of PDCD4 were described in the epidermis during wound healing, which suggest a potential role of PDCD4 in keratinocytes’ migration, proliferation and epidermal maturation.

### 2.8. Discussion

Prior work has documented the important role of PDCD4 as a tumor repressor in oncogenesis of various tumors. In the experimental oncology of skin, PDCD4 was identified as an important transformation suppressor of cutaneous tumor by regulating the susceptibility to tumor promotion through multiple molecular mechanisms [2,3,4]. However, the functional significance of PDCD4 in normal cutaneous biology and its exact role during epidermal tumorigenesis are still unclear. In this study, for the first time, we report that tumor suppressor PDCD4 is uniquely induced in a cell density-dependent manner in keratinocytes. Functional studies reveal that PDCD4 serves as an important regulator of keratinocytes’ proliferation and contact inhibition *in vitro*. Furthermore, the expression patterns of PDCD4 in normal skin, different hair cycles and the process of wound healing are described in detail *in vivo*, which suggests a steady-state regulating role of PDCD4 in epidermal homeostasis and wound healing.

As PDCD4 is a critical tumor suppressor in a variety of cancer development and down-regulation of PDCD4 is common feature of various tumors, PDCD4 expression regulation has been paid more attention [3,11,12,31]. Previous studies have demonstrated that PDCD4 could be regulated post-translationally by ubiquitin-proteasome system through Akt and ERK signaling pathway [3,12]. At post-transcriptional level, PDCD4 could be down-regulated by microRNAs miR-21 in many models [9,10,11]. The latest study shows that ZBP-89 can regulate PDCD4 expression at transcription level by direct binding its promoter [31]. These findings indicate that PDCD4 expression is regulated at multiple levels. By chance, we found that PDCD4 protein expression strongly increased in HaCaT keratinocytes upon achieving confluence. Furthermore, we demonstrated that the induction of PDCD4 protein in a cell density-dependent manner in keratinocytes was in accord with the increased mRNA level. It seems that the induction of PDCD4 in high cell density is one unique character of keratinocytes like another molecular HIF-1α [25]. Thus, for the first time, we demonstrated that PDCD4 is uniquely transcriptionally induced in a cell density-dependent manner in keratinocytes. As ZBP-89 and Sp transcription factor family members are important for PDCD4 transcription [31], their functions in regulating PDCD4 induction at higher keratinocytes density merit further study.

It is reported that cell density-dependent increased genes are always associated with growth arrest or contact inhibition [25,26]. This seems to imply that PDCD4 may play a role in regulating keratinocytes’ proliferation. We found that knockdown of PDCD4 by two specific siRNAs in HaCaT keratinocytes could obviously promote its proliferation in lower cell density and partially impair contact inhibition in confluent cells. These results were in agreement with the previous studies that PDCD4 played an essential role in proliferation of human carcinoma cells and rat vascular smooth muscle cells [20,29,32]. We propose that PDCD4 may reduce the susceptibility of mice to tumor formation in the skin carcinogenesis model by inhibiting keratinocytes’ proliferation and maintaining cell contact inhibition. Taken together, the function of PDCD4 in regulating keratinocytes proliferation and contact inhibition combined with its unique induction pattern indicate PDCD4 may act as important regulator of epidermal homeostasis, just like the well-known signaling pathway such as PI3K-Akt and TGF-β-Smad [33,34].

To investigate the role of PDCD4 in epidermal homeostasis, we examined the expression patterns of PDCD4 in epidermis by immunohistochemistry. Recently, PDCD4 have been identified with different sublocalization in epidermis of human and mouse [4,24]. In our study, these different expression patterns were further confirmed and the details of PDCD4 expression in mouse epidermis were also described. In the basal part of interfollicular epidermis, we found that PDCD4 was stained discontinuously in the basal epidermal cells. In the anagen hair follicle, PDCD4 is strongly induced compared with telogen hair follicle, and there is an obvious mutual inverse expression of PDCD4 and Ki67. We speculate that the induced expression of PDCD4 in anagen hair follicle *in vivo* may be similar to the upregulated PDCD4 in high cell-density of keratinocytes *in vitro*, which suggest PDCD4 as an important regulator of hair cycle by modulating keratinocytes’ proliferation. A previous report showed that PDCD4 transgenic mice exhibited the neonatal short-hair phenotype as determined by the complete regression of hair follicles into the upper dermis [4], which suggest that excessive PDCD4 could inhibit hair follicle cycle transition and support strongly our inference. In brief, the expression patterns of PDCD4 in mouse epidermis are described in detail, which suggest that PDCD4 is closely associated with epidermal homeostasis.

It is gradually accepted that common cellular and molecular mechanisms are active in wounds and in cancer tissue [35,36]. During reepithelialization of skin wounds, keratinocytes undergo a dramatic phenotypic conversion to become hyperproliferative, migratory, and invasive [37,38]. This transient healing response phenotypically resembles malignant transformation of keratinocytes during squamous cell carcinoma progression, which has been verified by laser capture microdissection-based *in vivo* genomic profiling of wound keratinocytes [39]. Similarly, recently, we reported that the important oncogenic microRNA miR-21 is upregulated postwound and plays a crucial role during cutaneous wound healing by regulating reepithelialization, wound contraction and collagen deposition [40]. However, wound can not induce tumor formation in normal conditions [41], which indicates that the wound healing process is self-limiting. Logically, the self-limiting of wound healing is partly due to the function of tumor suppressor genes. Thus, we proposed that the tumor suppressor PDCD4 may contribute to this self-limiting, and then we examined the expression patterns of PDCD4 during epidermal wound healing. As expected, we detected downregulated PDCD4 in the hyperproliferative and migratory epidermis as early as three days after wound, which showed similarity to the expression patterns of PDCD4 in many tumors [12,29,42]. While, PDCD4 became upregulated in the up stratum of hyperproliferative epidermis in the seven-day wounds, and similar expression patterns were also detected in the 10-day, 16-day and even 25-day wounds. These expression patterns of PDCD4 during reepithelialization revealed that PDCD4 may restrict the hyperproliferative epidermis to implement the epidermal self-limiting during wound repair.

In addition, it is to be noted that the intracellular translocation of endogenous PDCD4 between nucleus and cytoplasm were demonstrated in epidermal cells in both *in vitro* and *in vivo* models. In the HaCaT keratinocyte cells, we found that PDCD4 translocated from nucleus to cytoplasm upon serum starvation and Akt inhibition, which suggested the intracellular translocation of PDCD4 is likely to be a general mechanism [13,15,29]. In the normal and wounded epidermis, we found that PDCD4 translocated from cytoplasm to nucleus upon skin injury. As Akt phosphorylation is important for both skin wound healing and PDCD4 nucleic localization, we proposed that Akt phosphorylation upon wound healing leads to intracellular translocation of PDCD4. The different intracellular localization of PDCD4 in epidermal cells suggests PDCD4 may have different regulatory functions under different conditions.

## 3. Materials and Methods

### 3.1. Cell Culture

Human immortalized keratinocytes HacaT cells were maintained in RPMI-1640 medium (Gibco, Carlsbad, CA, USA). HEKn primary human neonatal epidermal keratinocytes (Lifeline Cell Technology, Walkersville, MD, USA) were maintained in EpiLife medium. A431 human squamous carcinoma, HEK-293 human embryonic kidney, HeLa human cervical carcinoma, and HepG2 human hepatoma cell-lines were cultured in Dulbecco’s modified Eagle’s medium. All culture media were supplemented with 10% fetal bovine serum, 100 units/mL of penicillin G and 100 μg/mL of streptomycin. Cells were grown in a 5% CO_2_ atmosphere at 37 °C in incubator. When plating cells 1 day before experiments, cell numbers were adjusted to reach 20%–100% of confluence at harvest. Briefly, to achieve 100% confluence of HaCaT cells for one day culture, 6 × 10^6^ cells were seeded into one 100 mm dish in 10 mL medium. By that analogy, seeding 1.2 × 10^6^ cells could get about 20% density after one day culture. After taking 5 photos per dish, the cell density would be calculated by Image J. Then, cells were used for experiments.

### 3.2. Animals and Wound Model of Skin

Male adult BALB/c mice, 8-week-old, 20–22 g body weight, were obtained from the Center of Experimental Animal, the Third Military Medicine University (TMMU, Chongqing, China). Mice were anesthetized with 1% Pentobarbital (30 mg/kg body weight) and had hair on the back shaved. One circular full-thickness skin excisions of 10 mm in diameter in the middle of back were aseptically created. Mice were sacrificed at different time points (3, 7, 10, 16, and 25 days) after skin injury. Then skin samples were harvested and fixed in 4% paraformaldehyde. The experiments were conducted in accord with the Guidelines for the Care and Use of Laboratory Animals of TMMU, and the experimental protocols used in this study were approved by the Animal Care Committee of TMMU.

### 3.3. Hair Cycle Induction

Hair follicle regeneration in the back skin of 6–8 weeks old mice was induced by depilation as described [43]. The depilation procedure can induce the highly synchronized hair follicle cycling, which is morphologically indistinguishable from spontaneous hair follicle cycling [44].

### 3.4. Western Blot Analysis

Total proteins from cells at different densities or cytosolic and nuclear fractions of total cellular proterin (NE-PER Nuclear and Cytoplasm Extraction Reagents, Life Tech, Carisbad, CA, USA) were extracted, resolved by SDS-PAGE and transferred onto PVDF membrane. Immune complexes were visualized with the ECL system, followed by exposure to X-ray film. The antibodies used in this study were listed as following: rabbit anti-PDCD4 antibody (CST, Beverly, MA, USA, 1:1000), rabbit anti-cyclin D1 antibody (Santa, Santa Cruz, CA, USA ,1:300), rabbit anti-Histone H3 antibody (CST, 1:1000), mouse anti-β-actin antibody (Beyotime, Hangzhou, China, 1:400). The relevant band intensities were quantified using Quantity One (Bio-Rad, Laboratories, Inc., Hercules, CA, USA)

### 3.5. Real-Time RT-PCR

Real-time PCR was performed with iQ5 system (Bio-Rad) using a SYBR Green assay. The expression values were normalized to β actin mRNA levels. Primer pairs used were listed as following: Human PDCD4: 5′-ATGAGCACAACTGATGTGGAAA-3′ and 5′-ACAGCTCTAGCAATAAACTGGC-3′. Human β-actin: 5′-TCCCTGGAGAAGAGCTACGA-3′ and 5′-AGCACTGTGTTGGCGTACAG-3′.

### 3.6. RNA Transfection

SiRNAs for PDCD4 have previously been described and the target sequences were as follows: siPDCD4-1 CAUUCAUACUCUGUGCUGG, siPDCD4-2 CACCAAUCAUACAGGAAUA [17]. SiRNAs (GenePharma, Shanghai, China) transfections were carried out with Lipofectamine 2000 (Invitrogen, Carlsbad, CA, USA) at the final concentration of 50 nmol/L.

### 3.7. Cell Proliferation Assay

For cell proliferation assays, HacaT cells were seeded 96-well plates at 2000 cells/well. After 24 h, cells were transfected with PDCD4 siRNAs and negative control at a concentration of 50 nM. Cell growth was measured at indicated times using the MST-8 assay with a Cell Counting Kit-8 (Dojin Laboratories, Mashiki, Japan). Absorbance was measured at 450 nm. At 5 days after transfection, cells were fixed in 4% paraformaldehyde and stained in a 0.1% crystal violet solution for visual display.

### 3.8. EdU Incorporation Assay

To investigate the role of PDCD4 in contact inhibition, about 80% confluent HaCaT cells in 96-well plates were transfected with PDCD4 siRNAs and negative control at a concentration of 50 nM. After 48 h, EdU (5-ethynyl-2’-deoxyuridine) incorporation assay of the confluent cells were determined by the Cell-Light™ EdU Apollo**^®^**567 In Vitro Imaging Kit (Ribobio Co., Guangzhou, China) according to the manufacturer’s protocol. Briefly, cells were incubated with 100 μL 50 μM EdU for 2 h before fixation, permeabilization, and EdU staining. Cell nuclei were stained with Hoechst 33342 at a concentration of 5 μg/mL for 30 min. The number of EdU-positive cells were counted under a fluorescent microscope in 10 fields with magnification of 200×.

### 3.9. Cell Cycle Analysis

HaCaT cells were plated in 6-well plates 1 day before experiments. When cells reached about 80% confluence, cells were transfected with PDCD4 siRNAs and negative control siRNA at a concentration of 50 nM. After 48 h, confluent cells were harvested and fixed in 75% ethanol at 4 °C overnight for subsequent cell cycle analysis by flow cytometer (Beckman Coulter, Miami, FL, USA).

### 3.10. Immunofluorescence

Cells were fixed in 4% paraformaldehyde and permeabilized in 0.1% NP40. Cells were stained for 2 h with PDCD4 (CST, 1:600), and staining detected by Cy3-conjugated secondary Ab (Beyotime, Hangzhou, Zhejiang, China). Cell nuclei were counterstained with 4’,6-diamidino-2-phenylindole (Roche, Basel, Switzerland).

### 3.11. Immnohistochemistry

Skin tissues were fixed in 4% paraformaldehyde, embedded in paraffin and sectioned serially at 5 μm. After deparaffinization, sections were stained with rabbit monoclonal anti-PDCD4 (CST, 1:100) and rabbit polyclonal anti-Ki67 (Abcam, CamBridge, UK, 1:200) antibodies. For human skin samples, AP (alkaline phosphatase)-Red systems were used for the final chromogen. For mouse skin samples, DAB (3,3′-Diaminobenzidine) chromogenic methods were used.

### 3.12. Statistical Analysis

All values were presented as mean ± SD. Statistical analysis was performed by one-way ANOVA, followed by Tukey’s multiple comparison tests. *p* < 0.05 was considered statistically significant.

## 4. Conclusions

Our results suggest that tumor suppressor PDCD4 is uniquely induced in a cell density-dependent manner in keratinocytes and serves as a regulator of keratinocyte cell proliferation and contact inhibition *in vitro*. Furthermore, enhanced expression of PDCD4 is detected in both anagen hair follicles and transient hyperproliferative wound epidermis *in vivo*, which suggests a steady-state regulating role of PDCD4 in epidermal homeostasis and wound healing.

## Supplementary Materials

Supplementary materials can be found at http://www.mdpi.com/1422-0067/17/1/8/s1.

## References

[1] Shibahara K., Asano M., Ishida Y., Aoki T., Koike T., Honjo T. (1995). Isolation of a novel mouse gene MA-3 that is induced upon programmed cell death. Gene.

[2] Cmarik J.L., Min H., Hegamyer G., Zhan S., Kulesz-Martin M., Yoshinaga H., Matsuhashi S., Colburn N.H. (1999). Differentially expressed protein PDCD4 inhibits tumor promoter-induced neoplastic transformation. Proc. Natl. Acad. Sci. USA.

[3] Schmid T., Jansen A.P., Baker A.R., Hegamyer G., Colburn N.H. (2008). Translation inhibitor PDCD4 is targeted for degradation during tumor promotion. Cancer Res..

[4] Jansen A.P., Camalier C.E., Colburn N.H. (2005). Epidermal expression of the translation inhibitor programmed cell death 4 suppresses tumorigenesis. Cancer Res..

[5] Chen Y., Knosel T., Kristiansen G., Pietas A., Garber M.E., Matsuhashi S., Ozaki I., Petersen I. (2003). Loss of PDCD4 expression in human lung cancer correlates with tumour progression and prognosis. J. Pathol..

[6] Afonja O., Juste D., Das S., Matsuhashi S., Samuels H.H. (2004). Induction of PDCD4 tumor suppressor gene expression by RAR agonists, antiestrogen and HER-2/neu antagonist in breast cancer cells. Evidence for a role in apoptosis. Oncogene.

[7] Yang H.S., Matthews C.P., Clair T., Wang Q., Baker A.R., Li C.C., Tan T.H., Colburn N.H. (2006). Tumorigenesis suppressor PDCD4 down-regulates mitogen-activated protein kinase kinase kinase kinase 1 expression to suppress colon carcinoma cell invasion. Mol. Cell. Biol..

[8] Mudduluru G., Medved F., Grobholz R., Jost C., Gruber A., Leupold J.H., Post S., Jansen A., Colburn N.H., Allgayer H. (2007). Loss of programmed cell death 4 expression marks adenoma-carcinoma transition, correlates inversely with phosphorylated protein kinase B, and is an independent prognostic factor in resected colorectal cancer. Cancer.

[9] Asangani I.A., Rasheed S.A., Nikolova D.A., Leupold J.H., Colburn N.H., Post S., Allgayer H. (2008). MicroRNA-21 (miR-21) post-transcriptionally downregulates tumor suppressor PDCD4 and stimulates invasion, intravasation and metastasis in colorectal cancer. Oncogene.

[10] Lu Z., Liu M., Stribinskis V., Klinge C.M., Ramos K.S., Colburn N.H., Li Y. (2008). MicroRNA-21 promotes cell transformation by targeting the programmed cell death 4 gene. Oncogene.

[11] Wang T., Zhang L., Shi C., Sun H., Wang J., Li R., Zou Z., Ran X., Su Y. (2012). TGF-β-induced miR-21 negatively regulates the antiproliferative activity but has no effect on EMT of TGF-β in HaCaT cells. Int. J. Biochem. Cell Biol..

[12] Dorrello N.V., Peschiaroli A., Guardavaccaro D., Pagano M. (2006). S6K1- and bTRCP-mediated degradation of PDCD4 promotes protein translation and cell growth. Science.

[13] Bohm M., Sawicka K., Siebrasse J.P., Brehmer-Fastnacht A., Peters R., Klempnauer K.H. (2003). The transformation suppressor protein PDCD4 shuttles between nucleus and cytoplasm and binds RNA. Oncogene.

[14] Fassan M., Pizzi M., Giacomelli L., Mescoli C., Ludwig K., Pucciarelli S., Rugge M. (2011). PDCD4 nuclear loss inversely correlates with miR-21 levels in colon carcinogenesis. Virchows Arch..

[15] Palamarchuk A., Efanov A., Maximov V., Aqeilan R.I., Croce C.M., Pekarsky Y. (2005). Akt phosphorylates and regulates PDCD4 tumor suppressor protein. Cancer Res..

[16] Yang H.S., Knies J.L., Stark C., Colburn N.H. (2003). PDCD4 suppresses tumor phenotype in JB6 cells by inhibiting AP-1 transactivation. Oncogene.

[17] Bitomsky N., Wethkamp N., Marikkannu R., Klempnauer K.H. (2008). siRNA-mediated knockdown of PDCD4 expression causes upregulation of p21(WAF1/Cip1) expression. Oncogene.

[18] Yang H.S., Jansen A.P., Komar A.A., Zheng X., Merrick W.C., Costes S., Lockett S.J., Sonenberg N., Colburn N.H. (2003). The transformation suppressor PDCD4 is a novel eukaryotic translation initiation factor 4A binding protein that inhibits translation. Mol. Cell. Biol..

[19] Yang H.S., Cho M.H., Zakowicz H., Hegamyer G., Sonenberg N., Colburn N.H. (2004). A novel function of the MA-3 domains in transformation and translation suppressor PDCD4 is essential for its binding to eukaryotic translation initiation factor 4A. Mol. Cell. Biol..

[20] Guo X., Li W., Wang Q., Yang H.S. (2011). AKT Activation by PDCD4 knockdown up-regulates cyclin D1 expression and promotes cell proliferation. Genes Cancer.

[21] Ozpolat B., Akar U., Steiner M., Zorrilla-Calancha I., Tirado-Gomez M., Colburn N., Danilenko M., Kornblau S., Berestein G.L. (2007). Programmed cell death-4 tumor suppressor protein contributes to retinoic acid-induced terminal granulocytic differentiation of human myeloid leukemia cells. Mol. Cancer Res..

[22] Zhang H., Ozaki I., Mizuta T., Hamajima H., Matsuhashi S. (2006). Involvement of programmed cell death 4 in transforming growth factor-β1-induced apoptosis in human hepatocellular carcinoma. Oncogene.

[23] Santhanam A.N., Baker A.R., Hegamyer G., Kirschmann D.A., Colburn N.H. (2010). PDCD4 repression of lysyl oxidase inhibits hypoxia-induced breast cancer cell invasion. Oncogene.

[24] Matsuhashi S., Narisawa Y., Ozaki I., Mizuta T. (2007). Expression patterns of programmed cell death 4 protein in normal human skin and some representative skin lesions. Exp. Dermatol..

[25] Cho Y.S., Bae J.M., Chun Y.S., Chung J.H., Jeon Y.K., Kim I.S., Kim M.S., Park J.W. (2008). HIF-1α controls keratinocyte proliferation by up-regulating p21(WAF1/Cip1). Biochim. Biophys. Acta.

[26] Swat A., Dolado I., Rojas J.M., Nebreda A.R. (2009). Cell density-dependent inhibition of epidermal growth factor receptor signaling by p38α mitogen-activated protein kinase via Sprouty2 downregulation. Mol. Cell. Biol..

[27] Belso N., Szell M., Pivarcsi A., Kis K., Kormos B., Kenderessy A.S., Dobozy A., Kemeny L., Bata-Csorgo Z. (2008). Differential expression of D-type cyclins in HaCaT keratinocytes and in psoriasis. J. Investig. Dermatol..

[28] Lankat-Buttgereit B., Goke R. (2009). The tumour suppressor PDCD4: Recent advances in the elucidation of function and regulation. Biol. Cell.

[29] Wei N., Liu S.S., Chan K.K., Ngan H.Y. (2012). Tumour suppressive function and modulation of programmed cell death 4 (PDCD4) in ovarian cancer. PLoS ONE.

[30] Jones P.H., Simons B.D., Watt F.M. (2007). Sic transit gloria: Farewell to the epidermal transit amplifying cell?. Cell Stem Cell.

[31] Leupold J.H., Asangani I.A., Mudduluru G., Allgayer H. (2012). Promoter cloning and characterization of the human programmed cell death protein 4 (*PDCD4*) gene: Evidence for ZBP-89 and Sp-binding motifs as essential PDCD4 regulators. Biosci. Rep..

[32] Liu X., Cheng Y., Yang J., Krall T.J., Huo Y., Zhang C. (2010). An essential role of PDCD4 in vascular smooth muscle cell apoptosis and proliferation: Implications for vascular disease. Am. J. Physiol. Cell Physiol..

[33] Pankow S., Bamberger C., Klippel A., Werner S. (2006). Regulation of epidermal homeostasis and repair by phosphoinositide 3-kinase. J. Cell Sci..

[34] Cui W., Fowlis D.J., Cousins F.M., Duffie E., Bryson S., Balmain A., Akhurst R.J. (1995). Concerted action of TGF-β1 and its type II receptor in control of epidermal homeostasis in transgenic mice. Genes Dev..

[35] Dvorak H.F. (1986). Tumors: Wounds that do not heal. Similarities between tumor stroma generation and wound healing. N. Engl. J. Med..

[36] Schafer M., Werner S. (2008). Cancer as an overhealing wound: An old hypothesis revisited. Nat. Rev. Mol. Cell Biol..

[37] Martin P. (1997). Wound healing—Aiming for perfect skin regeneration. Science.

[38] Singer A.J., Clark R.A. (1999). Cutaneous wound healing. N. Engl. J. Med..

[39] Pedersen T.X., Leethanakul C., Patel V., Mitola D., Lund L.R., Dano K., Johnsen M., Gutkind J.S., Bugge T.H. (2003). Laser capture microdissection-based *in vivo* genomic profiling of wound keratinocytes identifies similarities and differences to squamous cell carcinoma. Oncogene.

[40] Wang T., Feng Y., Sun H., Zhang L., Hao L., Shi C., Wang J., Li R., Ran X., Su Y. (2012). miR-21 regulates skin wound healing by targeting multiple aspects of the healing process. Am. J. Pathol..

[41] Wong S.Y., Reiter J.F. (2011). Wounding mobilizes hair follicle stem cells to form tumors. Proc. Natl. Acad. Sci. USA.

[42] Wang Q., Sun Z., Yang H.S. (2008). Downregulation of tumor suppressor PDCD4 promotes invasion and activates both β-catenin/Tcf and AP-1-dependent transcription in colon carcinoma cells. Oncogene.

[43] Paus R., Stenn K.S., Link R.E. (1990). Telogen skin contains an inhibitor of hair growth. Br. J. Dermatol..

[44] Muller-Rover S., Handjiski B., van der Veen C., Eichmuller S., Foitzik K., McKay I.A., Stenn K.S., Paus R. (2001). A comprehensive guide for the accurate classification of murine hair follicles in distinct hair cycle stages. J. Investig. Dermatol..

