# Supplementary material for: Cell Density-Dependent Upregulation of PDCD4 in Keratinocytes and Its Implications for Epidermal Homeostasis and Repair
Source: Int J Mol Sci. 2015 Dec 23;17(1):8. doi: 10.3390/ijms17010008 (PMC4730255; doi:10.3390/ijms17010008)
Supplement: Supplementary file 1 [file ijms-17-00008-s001.pdf]

Supplementary Materials: Cell Density-Dependent Upregulation of PDCD4 in Keratinocytes and Its Implications for Epidermal Homeostasis and Repair

Tao Wang, Shuang Long, Na Zhao, Yu Wang, Huiqin Sun, Zhongmin Zou, Junping Wang, Xinze Ran and Yongping Su

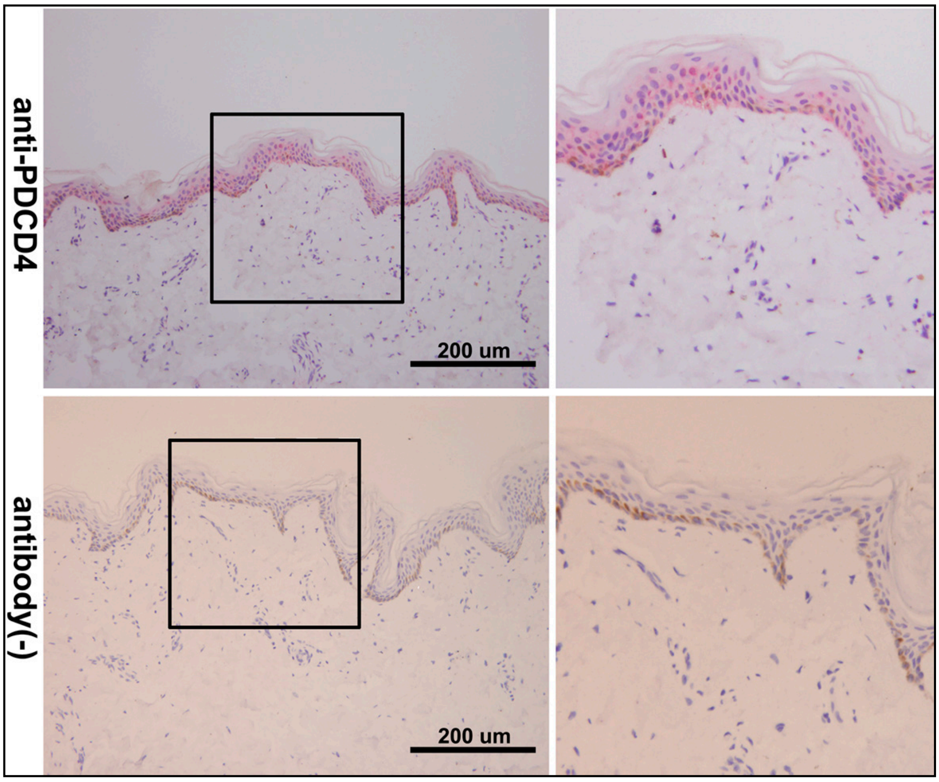

Figure S1. Immunohistochemical localization of PDCD4 in human skin. **Top**, PDCD4 was stained diffusely in the suprabasal cells and discontinuously in the basal cells featured by heterogeneous nucleus localization (detected by AP-Red system); **Bottom**, negative controls were presented. Of note, there were spontaneous yellow-stains in basal cells owing to pigmentation. The black boxes in the right panel show the sources of the enlarged detailed area of left panel.
